# The leucine-rich repeat domains of BK channel auxiliary γ subunits regulate their expression, trafficking, and channel-modulation functions

**DOI:** 10.1016/j.jbc.2022.101664

**Published:** 2022-01-30

**Authors:** Guanxing Chen, Qin Li, Jiusheng Yan

**Affiliations:** 1 Department of Anesthesiology and Perioperative Medicine, The University of Texas MD Anderson Cancer Center, Houston, Texas, USA; 2 Graduate Programs of Neuroscience and Biochemistry and Cell Biology, The University of Texas MD Anderson Cancer Center UT Health Graduate School of Biomedical Sciences, Houston, Texas, USA

**Keywords:** leucine-rich repeat, BK channel, regulation, auxiliary subunit, N-glycosylation, surface expression, DDM, dodecyl-beta-D-maltoside, ER, endoplasmic reticulum, LRR, leucine-rich repeat, LRRC, leucine-rich repeat–containing, TM, transmembrane

## Abstract

As high-conductance calcium- and voltage-dependent potassium channels, BK channels consist of pore-forming, voltage-, and Ca^2+^-sensing α and auxiliary subunits. The leucine-rich repeat (LRR) domain–containing auxiliary γ subunits potently modulate the voltage dependence of BK channel activation. Despite their dominant size in whole protein masses, the function of the LRR domain in BK channel γ subunits is unknown. We here investigated the function of these LRR domains in BK channel modulation by the auxiliary γ1–3 (LRRC26, LRRC52, and LRRC55) subunits. Using cell surface protein immunoprecipitation, we validated the predicted extracellular localization of the LRR domains. We then refined the structural models of mature proteins on the membrane via molecular dynamic simulations. By replacement of the LRR domain with extracellular regions or domains of non-LRR proteins, we found that the LRR domain is nonessential for the maximal channel-gating modulatory effect but is necessary for the all-or-none phenomenon of BK channel modulation by the γ1 subunit. Mutational and enzymatic blockade of N-glycosylation in the γ1–3 subunits resulted in a reduction or loss of BK channel modulation by γ subunits. Finally, by analyzing their expression in whole cells and on the plasma membrane, we found that blockade of N-glycosylation drastically reduced total expression of the γ2 subunit and the cell surface expression of the γ1 and γ3 subunits. We conclude that the LRR domains play key roles in the regulation of the expression, cell surface trafficking, and channel-modulation functions of the BK channel γ subunits.

The large-conductance, voltage- and calcium-activated potassium (BK, or K_Ca1.1_) channels are ubiquitously expressed and critically involved in various cellular and physiological processes, such as regulation of neuron firing and transmission (1, 2), motor coordination (3), learning and memory (4, 5), circadian rhythmicity (6, 7), the contractile tone of almost all types of smooth muscle cells (8, 9), and resting K^+^ efflux in secretory epithelial cells (10, 11). BK channel function is regulated by various auxiliary β and γ subunits and LINGO1 that confer tissue-specific gating and pharmacological properties (12, 13, 14, 15).

The four BK channel γ subunits are a group of leucine-rich repeat (LRR)–containing (LRRC) membrane proteins that consist of the γ1 (LRRC26), γ2 (LRRC52), γ3 (LRRC55), and γ4 (LRRC38) subunits (16, 17). The BK channel γ subunits are expressed in different tissues and play important physiological roles. The γ1 subunit is highly expressed in the salivary glands, prostate, and trachea and is moderately present in thyroid gland, thymus, colon, aorta, and fetal brain; γ2 is predominantly expressed in the testes; and γ3 is primarily expressed in the nervous system (17). The γ1 subunit confers an unusual capability to the BK channel, making it active in the absence of calcium under the cells’ resting state (16, 17), and is thus able to play an important role in resting K^+^ efflux and fluid secretion in nonexcitable secretory cells (10, 11). The γ2 subunit potently regulates BK channel function in cochlear inner hair cells (18).

The BK channel γ subunits have a characteristic LRR domain, a single transmembrane (TM) segment, and a short intracellular C-terminal tail. The γ subunits display an exceptionally large range of capabilities in shifting the BK channel’s voltage dependence of activation toward the hyperpolarizing direction by approximately 145 (BKγ1), 105 (BKγ2), and 50 mV (BKγ3) in the absence of intracellular Ca^2+^ (Table S1). This is in contrast to the complex effects and mechanisms of the four double membrane–spanning β subunits on many aspects of BK channel gating (19, 20, 21, 22, 23, 24, 25, 26). Furthermore, the γ1 subunit possesses an uncommon binary “all-or-none” phenotype in that the voltage dependence of BK channel gating was either fully shifted to the negative voltage direction or unchanged when the expression of the γ1 subunit was limited (27). This is in contrast to the commonly observed incremental modulatory effects of most other K^+^ channel auxiliary proteins, such as the BKβ subunits on BK channels (28), KCNE on KCNQ channels (29, 30), and KChiP on Kv4 channels (31). In the classic model, most auxiliary subunits bind to the channel with 4-fold symmetry to mirror the pore-forming α subunit’s 4-fold structural symmetry; thus, the channel-regulatory effect is expected to be incremental upon variation in the relative molecular ratio of the auxiliary subunit to core subunit. Interesting, up to four γ1 subunit molecules can bind to a single BK channel (32, 33, 34); however, a single γ1 subunit molecule per channel was considered to be enough to produce the full modulatory effect (32).

The cryo-EM structures of the human BK channel in complex with the β4 subunit (35) provide structural insight into the mechanisms of channel modulation by β subunits. However, our understanding of the mechanisms of BK channel regulation by the auxiliary γ subunits remains very limited. We previously reported that the BK channel modulatory functions of different γ subunits are determined by their single TM segment and the adjacent positively charged amino acids on the C-terminal side (36). Nevertheless, the function of the characteristic LRR domains of the γ subunits remains largely unknown.

There are hundreds of LRRC proteins in the human protein database. Since the identification of the LRRC-type BKγ subunit (16, 17), an increasing number of LRRC proteins have been found to function as regulatory or core proteins of ion channels, including LINGO1 for BK channels (15), LRRC52 (BKγ2) for the Slo3 channel (37), AMIGO for Kv2.1 (38), LRRC10 and LRRK2 for voltage-gated Ca^2+^ channels (39, 40), nyctalopin for TRPM1 (41), and LRRC8A-E for volume-regulated anion channels (42, 43). However, currently, little is known about the roles of their LRR domains in ion channel regulation.

All BK channel γ subunits contain single or multiple canonical consensus (N-X-S/T) N-linked glycosylation sites in their LRR domains. So far, only the γ1 subunit has been identified to be glycosylated at the N147 site, but its function has not been explored (17). Membrane proteins with large extracellular regions can be N-glycosylated during their synthesis and processing. In the trafficking pathway, a mannose-rich glycan precursor is covalently linked to the specific Asn (N) residues on the extracellular domain or region of the nascent protein in the endoplasmic reticulum (ER) lumen. The N-linked precursor glycan is first modified by trimming as proteins exit the ER and then by sequential addition of diverse monosaccharides in the Golgi apparatus before they are targeted to their final destination. This N-glycosylation process results in substantial diversity of N-glycans and regulates membrane protein structure and function. N-glycosylation plays an important role in the regulation of ion channel proteins’ structure and function by affecting their folding, stability, trafficking, protein interactions, and biophysical or pharmacological properties (44).

In previous studies, we found that swapping the LRR domain in the γ1 subunit with that of γ2–4 subunits resulted in small 15- to 30-mV shifts in G-V toward the negative voltage direction (36), suggesting that they have functional similarities, in spite of some small difference in their impacts on BK channel voltage gating. In addition, deletion of the LRR domain or even the individual LRR units caused a full loss of the γ1 subunit’s channel modulation function (16), suggesting that the LRR domain has a role in the γ subunits’ expression, trafficking, or structures on the membrane. Therefore, in this study, we investigated structure and function of the LRR domains in the BK channel γ1–3 subunits. We determined the proteins’ membrane topologies with cell surface protein immunoprecipitation and investigated the LRR domains’ function with two new strategies by replacing the LRR domain with other types of extracellular domains that potentially facilitate proper cell surface expression and by altering the N-glycosylation statuses of the LRR domain. Our results confirmed the previous prediction of membrane topology and reveal the important roles of the LRR domains in the expression, trafficking, and all-or-none modulatory functions of BK channel auxiliary γ subunits.

## Results

### Structural models of BK channel γ subunits refined by molecular dynamic simulation

The BK channel γ subunits each contain an N-terminal short hydrophobic segment, an LRR domain, a single TM segment, and a short C-terminal tail (Fig. S1). We predicted the presence of an N-terminal signal peptide sequence, the extracellular location of the LRR domain, and the intracellular location of the C terminus (16, 17). We reported that the N-terminal short hydrophobic sequence is cleaved in the mature protein and in the BKα-γ fusion constructs in which the whole γ subunit sequences are fused to the C terminus of the BKα subunit (17). On the basis of our previously modeled structure, the γ subunits’ LRR domain comprises six LRR repeats (LRR1–6) in the middle, capped by two cysteine-rich LRRNT and LRRCT regions on the N-terminal and C-terminal sides, respectively (16). Each LRR repeat is composed of 22 to 25 (mostly 24) residues with a consensus sequence of xxLxxLxxxLxLxxxxLxLxxN for LRR1 and xLxxLxxxxLxxxx xLxxLxLxxN for LRR2–6 (where x can be any amino acid and L can be replaced by I, V, F, or Y) (Fig. S1). Both the LRRNT and LRRCT regions contain two pairs of cysteine residues that are predicted to form disulfide bridges.

**Figure 1:**
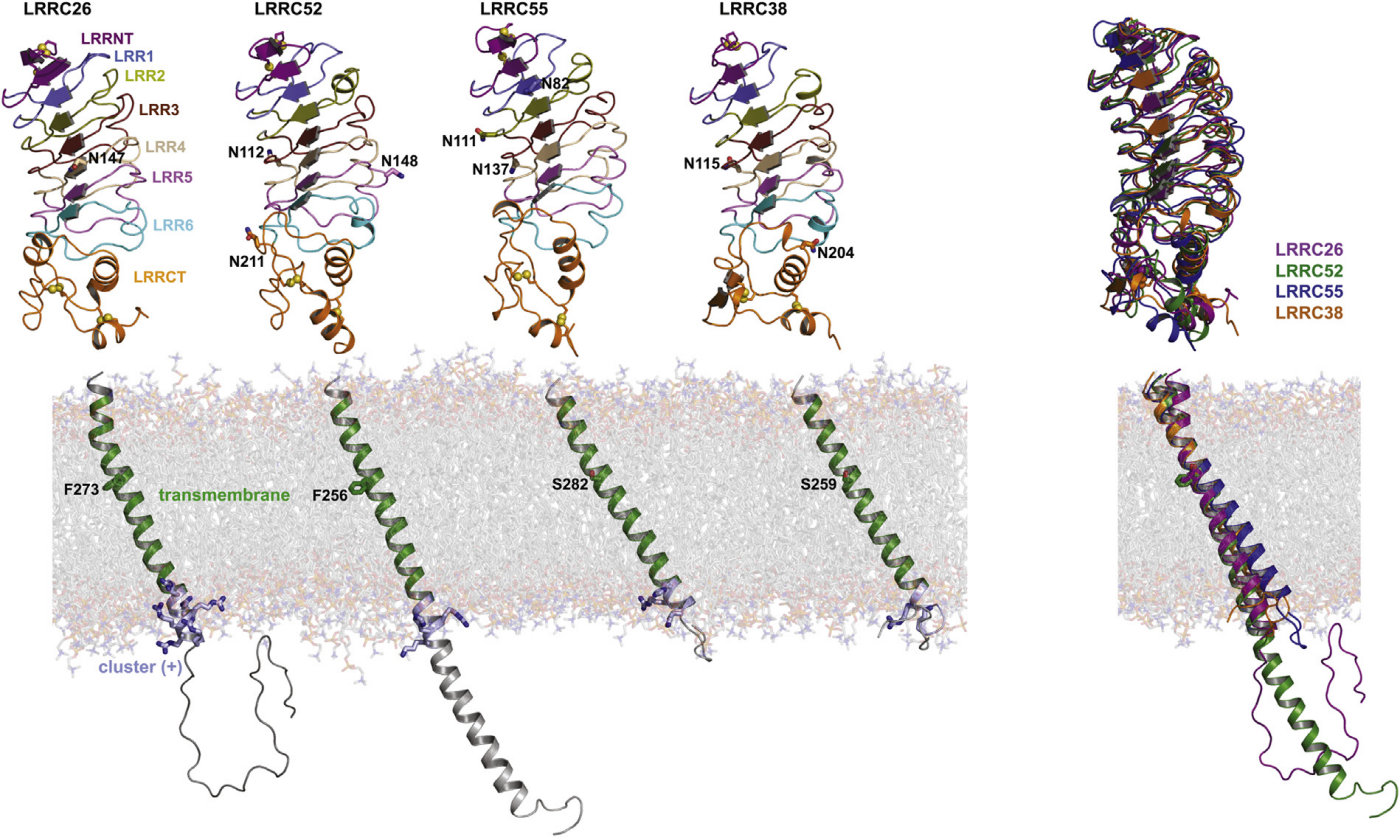
**Structural models of the BK channel γ subunits.** The structural models of LRR domains and the rest were obtained from the last frames of 100-ns MD simulation. For comparison, the LRR domains and TM domains among different γ subunits were aligned and displayed separately. For visualization purpose, TM domains after structural alignment were embedded manually on the same lipid bilayer.

The recently released protein structure database of AlphaFold (45) provides predicted structural models of the whole proteins. Given the structural conservation of the LRR domains across LRR proteins and the α-helix feature of the TM region, we consider the AlphaFold prediction to be reasonable and a good structural basis for further refinement. To refine the structural models, we removed the signal peptide sequences and added POPC lipid bilayer for the predicted TM region and performed molecular dynamic simulation in a water box containing 0.15 M KCl. In the simulated structures, the relative positions of the LRR domains to the membrane are largely variable. Therefore, to perform a structural comparison of the four γ subunits, we aligned and presented the LRR domains and the TM regions separately. In the modeled structures, the six stacked LRR repeats (LRR1–6) form a curved parallel β-sheet lining the concave face and mostly turns or occasionally helices flanking the convex circumference (Fig. 1). The conserved hydrophobic residues (mostly Leu) point inward and form the hydrophobic core of the LRR domain. The LRRNT stabilizes the LRR domain on the N-terminal side by contributing two additional β-strands (one antiparallel and one parallel) and forming two disulfide bridges. The LRRCT contains helical structures and two disulfide bridges. The LRR repeats are largely similar, whereas the LRRCT regions are less conserved and more variable in structure among the four γ subunits. The TM segments are helical and tilted in the membrane. We had found that the γ1 and γ2 subunits’ TM segments account for an ∼100-mV shift in the voltage dependence of BK channel activation, whereas the γ3 and γ4 subunits’ TM segments do not contribute (46). Of interest, the TM segments of the γ1 and γ2 subunits are slightly bent in the middle compared with those of the γ3 and γ4 subunits (Fig. 1). This is likely caused by the difference in the position of residue 273 (γ1 sequence), which is a Phe in γ1 and γ2 but a Ser in γ3 and γ4. We found that the F↔S switch at this position plays a key role in differentiating the function of the γ1–4 subunits’ single TM segment in BK modulation (46). The TM-adjacent positively charged clusters are largely α-helical structures (Fig. 1). The C tails, except that in the γ2 subunit, are mostly disordered in structure. We have not yet identified a role for C tails beyond the cluster regions in the γ subunits’ function on BK channel modulation.

### Membrane topology of BK channel γ subunits

**Figure 2:**
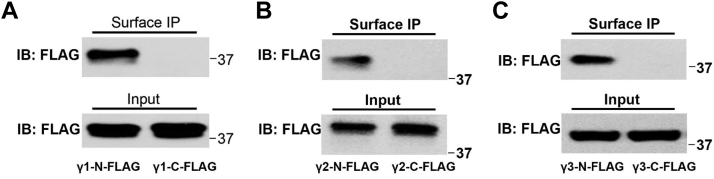
**Surface immunoprecipitation analyses of membrane topology of BK channel γ subunits.***A*–*C*, immunoblot analyses of FLAG-tagged γ1 (*A*), γ2 (*B*), and γ3 (*C*) in samples of surface immunoprecipitation (*upper panel*s) and total protein (whole-cell lysate; *bottom panels*). IB, immunoblot; IP, immunoprecipitation.

To validate the predicted membrane topology of the BK channel γ subunits, we developed a cell surface protein immunoprecipitation method to detect the subunits’ expression on plasma membranes. We introduced the FLAG peptide tag to the γ subunits’ N termini, located after the cleaved signal peptides and immediately in front of the LRRNT unit and C termini, respectively. The cells that expressed the FLAG-tagged γ subunits were first probed by a rabbit anti-FLAG antibody via incubation of the live cells with the antibody and then immunoprecipitated upon membrane protein extraction from cell membranes with detergent. Immunoblotting of the immunoprecipitated samples with a mouse anti-FLAG antibody showed that only the N-terminally FLAG-tagged γ1, γ2, and γ3 proteins could be enriched, whereas those containing the C-terminal FLAG-tag were not detected (Fig. 2, *A*–*C*). Conversely, both N- and C-terminally tagged FLAG epitopes were similarly detected by immunoblotting of total proteins in whole-cell lysate (Fig. 2, *A*–*C*). Our results provide direct evidence that the BK channel γ subunits’ LRR domains are located on the extracellular side, while their short C-terminal tails are positioned on the intracellular side.

### Loss of LRR domain affected the all-or-none phenomenon of BK channel modulation by the γ1 subunit

**Figure 3:**
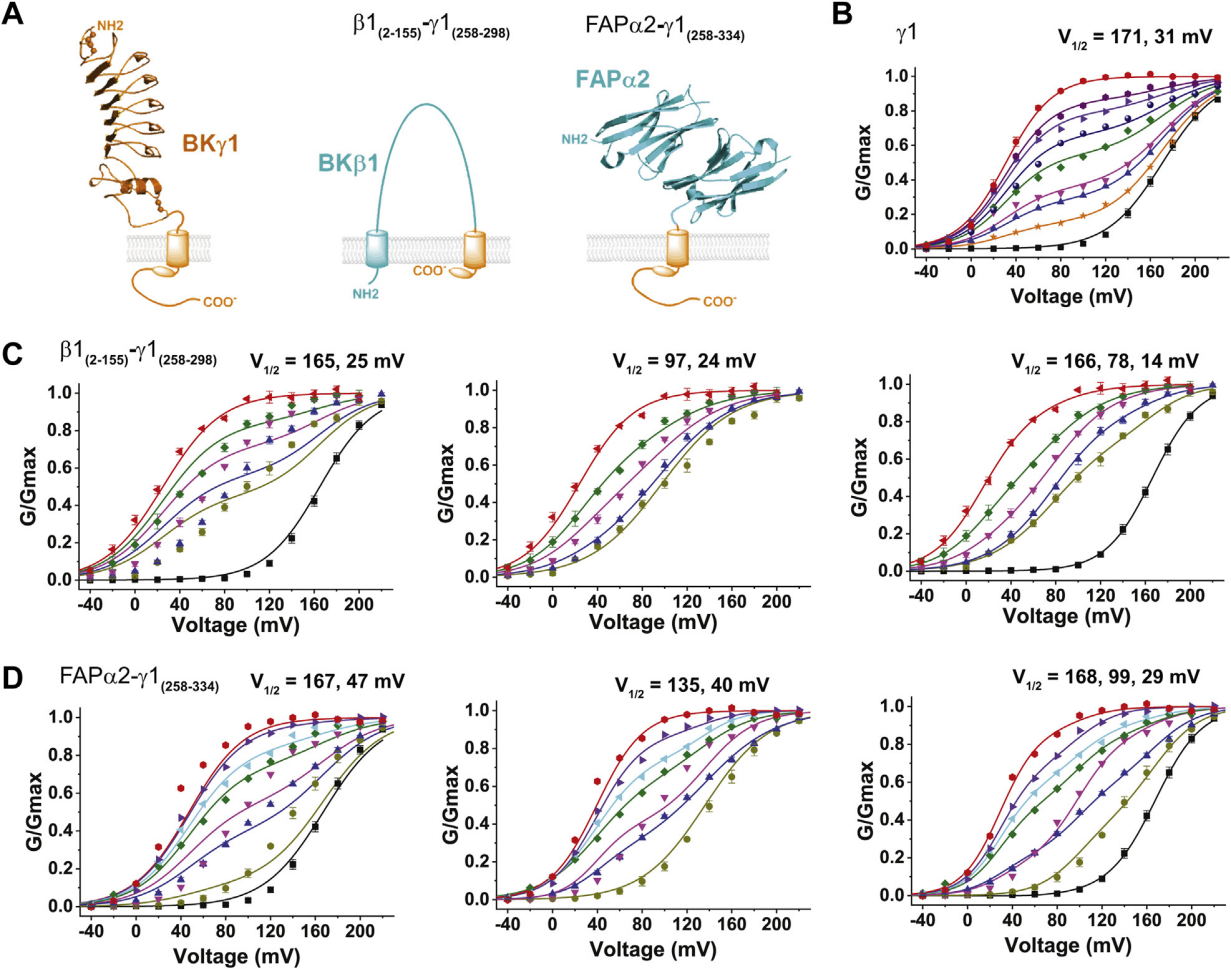
**The all-or-none phenomenon of BK channel modulation by the γ1 subunit was absent upon loss of its LRR domain.***A*, structural cartoons of the γ1 and its chimeric constructs. *B*, the G-V curves of the BK channels were best fitted with a double-Boltzmann function when the γ1 subunit’s expression is limited. *C*, double- and triple-Boltzmann function fittings of the G-V relationships of BK channels with limited expression of the β1_(2–155)_-γ1_(258–298)_ chimeric construct. *D*, double- and triple-Boltzmann function fittings of the G-V relationships of BK channels with limited expression of the FAPα2-γ1_(258–334)_ chimeric construct. The double- and triple-Boltzmann function fittings of all G-V relationships in a dataset were done in a global manner with shared V_1/2_ and z parameters. The best fitted V_1/2_ values (shown on *top*) were obtained after the fit was converged and a chi-square tolerance value of 1 × 10^−9^ was reached. All data were collected at 0 [Ca^2+^]_i_.

We previously reported a β1_(2–155)_-γ1_(258–298)_ chimeric construct in which the BK channel β1 subunit’s second transmembrane segment and the short intracellular C terminus are replaced by the γ1 subunit’s TM segment and the adjacent positively charged residue cluster (residues 258–298) (Fig. 3*A*) (46). The β1_(2–155)_-γ1_(258–298)_ chimeric protein when fused to the C terminus of the BKα subunit overall displayed a similar function of the γ1 subunit, with nearly no noticeable contribution from the β1 subunit to BK channel gating, supporting a transplantable function of the γ1 single TM and the adjacent charged cluster in BK channel modulation (46). In this study, we examined the function of this LRR domain–lacking chimeric protein on BK channel modulation by cotransfecting the plasmids of BKα and γ1 or β1_(2–155)_-γ1_(258–298)_ at a ratio of ∼1:1 or 2:1 (whole plasmid weight), a heterologous expression condition that is not optimal for BK channel modulation by the γ1 subunit. With the intact γ1 subunit, the obtained G-V relationship curves of BK channels showed some variation from cells to cells (Fig. 3*B*), presumably caused by a cell-to-cell variation in the absorption of different plasmid DNAs. However, the G-V curves were collectively well fitted by a double-Boltzmann function, an equation depicting the additive effects of two channel populations with distinct voltage dependence in channel activation. The fitting result showed two populations of channels, one fully modulated (V_1/2_ = 31 mV) and the other not modulated at all (V_1/2_ = 171 mV) (Fig. 3*B* and Table S2), in agreement with the known all-or-none modulatory effect of the γ1 subunit (27). The all-or-none phenomenon was not obvious with the β1_(2–155)_-γ1_(258–298)_ chimeric protein. The resulting G-V relationship curves, particularly those more shifted toward the positive voltage direction, cannot be fitted by a double-Boltzmann function using V_1/2_ values including the unmodulated channels (*e.g.*, 165 mV) (Fig. 3*C*, left panel). Without restriction on V_1/2_ values, the best double-Boltzmann fitting produced a low V_1/2_ value of the fully modulated channels and an intermediate V_1/2_ value of 97 mV (Fig. 3*C*, middle panel) that differs from the expected high V_1/2_ value of the fully unmodulated channels. Fitting with a triple-Boltzmann function produced a more reasonable result of three V_1/2_ values that can account for the fully modulated channels (14 mV) and the unmodulated channels (166 mV), and some intermediate state(s) of channel modulation (78 mV). Given that the β1_(2–155)_ part might complicate the interpretation because of the modulatory function of the β1 subunit on BK channels, we further evaluated the function of the LRR domain by replacing it with a distinct and BK channel-unrelated protein domain, called FAPα2 (SpectraGenetics, Inc) or L5-MG dimer (47). FAPα2 is an engineered extracellularly located protein domain that contains two copies of α-type fluorogen-activating peptide (SpectraGenetics, Inc), which is an antibody light chain variable domain that can bind malachite green dye to produce fluorescence (47). The resulting FAPα2-γ1_(258–334)_ construct (Fig. 3*C*) can still cause a large shift in G-V toward the negative voltage direction (Fig. 3*D*) that is comparable with that induced by the intact γ1 subunit. However, similar to what was observed with the β1_(2–155)_-γ1_(258–298)_ construct, the BK channel G-V relationships obtained with FAPα2-γ1_(258–334)_ construct is not consistent with the all-or-none phenomenon of BK channel modulation by the γ1 subunit. The double-Boltzmann function failed to produce reasonable fits for the G-V relationships if a high V_1/2_ value of the fully unmodulated channels was forced to be included in fitting (Fig. 3*D*, right panel). An intermediate V_1/2_ value of 135 or 99 mV was needed to produce the best fits with a double- or triple-Boltzmann function, respectively (Fig. 3*D*, middle and right panels). The necessity of an intermediate V_1/2_ value to fit the G-V relationship with either a double- or a triple-Boltzmann function implies the presence of one or more channel populations that are only partially modulated, *i.e.*, neither fully modulated nor unmodulated. These results with β1_(2–155)_-γ1_(258–298)_ and FAPα2-γ1_(258–334)_ constructs showed that the LRR domain is dispensable for the maximal shift in G-V curve induced by the γ1 subunit. However, the absence of the LRR domain in the γ1 subunit led to a loss of the all-or-none phenomenon of BK channel modulation by the γ1 subunit as some additional status of channel modulation appeared.

### Identification of N-glycosylation sites on LRR domains of the γ subunits

The functional analysis described above with the chimeric β1_(2–155)_γ1_(258–298)_ and FAPα2-γ1_(258–334)_ constructs also clearly showed that the LRR domain is not absolutely required for the maximal channel-modulatory effect of the γ1 subunit in terms of the shift in the voltage dependence of BK channel activation. However, deletion of the whole LRR domain or even an individual LRR unit in the γ1 subunit led to the full loss of the protein’s function in BK channel modulation (16), suggesting some other key role for the LRR domain in BK channel γ subunits.

**Figure 4:**
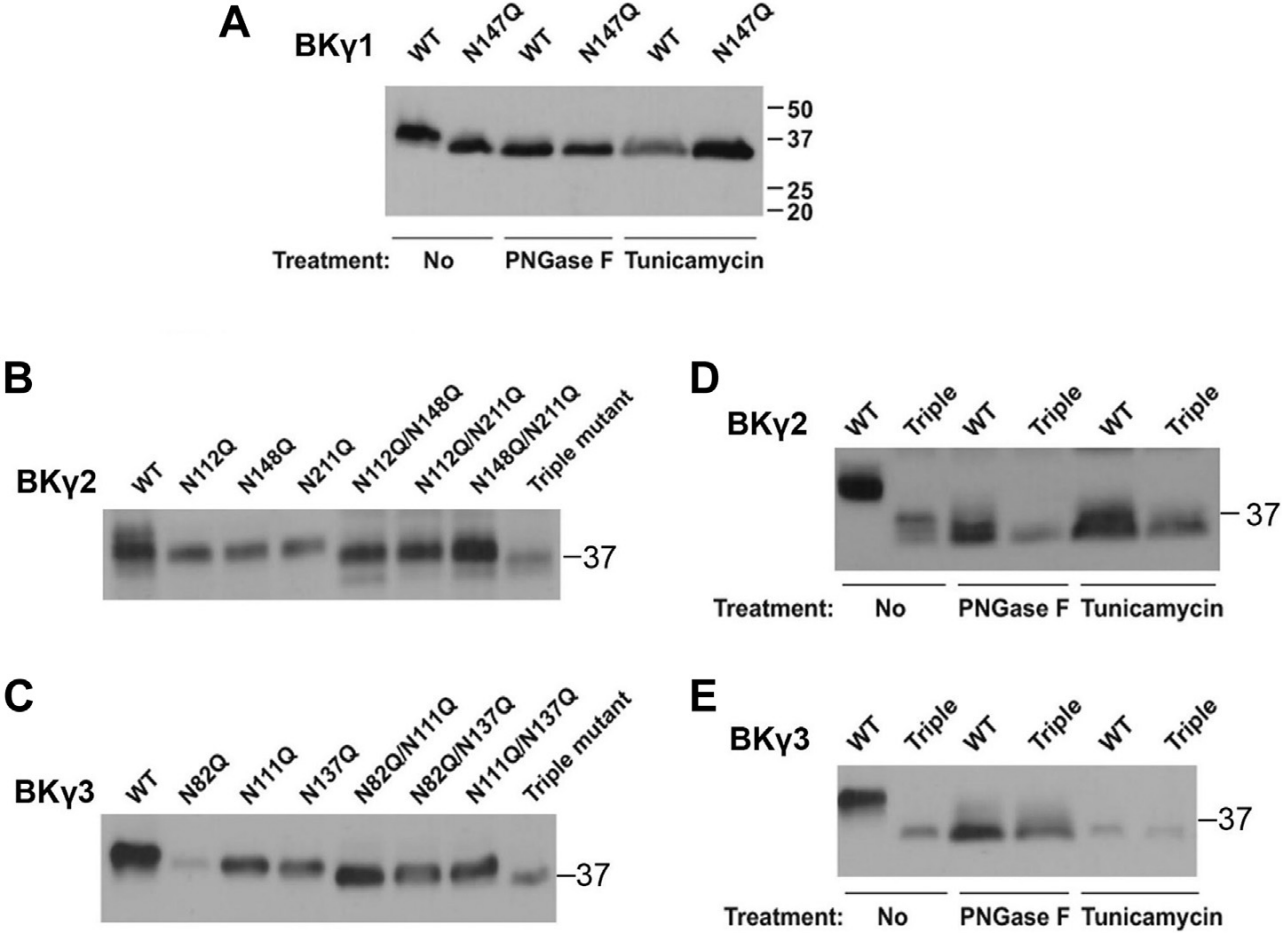
**N-linked glycosylation sites on the extracellular LRR domains of BK channel γ subunits.***A*, immunoblot analyses of whole-cell lysates from cells that were transiently transfected with γ1 WT and glycosylation-null (N→Q mutation) N147Q mutant, in the absence and presence of lysate treatment with PNGase F or cell treatment with tunicamycin. *B*, immunoblot analyses of whole-cell lysates from cells that were transiently transfected with γ2 WT and glycosylation-null single-, double-, and triple-site mutants at N112, N148, and N211 sites. *C*, immunoblot analyses of whole-cell lysates from cells transiently transfected with γ3 WT and glycosylation-null single-, double-, and triple-site mutants at N82, N111, and N137 sites. *D*, immunoblot analyses of whole-cell lysates from cells transiently transfected with γ2 WT and glycosylation-null triple-site mutant in the absence and presence of lysate treatment with PNGase F or cell treatment with tunicamycin. *E*, immunoblot of whole-cell lysates from cells transiently transfected with γ3 WT and glycosylation-null triple-site mutant in the absence and presence of lysate treatment with PNGase F or cell treatment with tunicamycin. For all immunoblot analyses, the γ1–3 proteins were all FLAG-tagged at the C termini and probed with an anti-FLAG antibody.

To further understand the function of the LRR domains in the BK channel γ subunits, we investigated their posttranslational modifications by N-glycosylation. Manipulation of their N-glycosylation status allowed us to perturb the LRR domain’s structure and function in a site-specific manner, while the overall protein structures of the γ subunits were largely maintained. Consistent with the results of our previous report (17), mutation of the predicted N-glycosylation site N147 (Figs. S1 and 1) to Q on the γ1 LRR domain resulted in a slightly reduced molecular size on SDS-PAGE. A similar change in γ1 protein migration on SDS-PAGE was observed after the enzymatic removal of the N-linked glycan via treatment of the cell lysate with peptide-N-glycosidases F (PNGase F) or by inhibiting the N-linked glycosylation process via treatment of cells with tunicamycin (Fig. 4*A*), an inhibitory analog of the substrate of GlcNAc-1-P-transferase, which catalyzes the first and committed step of N-linked glycosylation in the ER membrane. These results suggest that N147 is the only N-glycosylation site in the γ1 subunit.

Similarly, we generated a series of N-glycosylation blockade mutants in the γ2 and γ3 subunits at their consensus sites (Figs. S1 and 1). We mutated the residues N112, N148, and N211 in the γ2 subunit and N82, N111, and N137 in the γ3 subunit to Q and generated single-, double-, and triple-site N-glycosylation blockade mutants, respectively. Using SDS-PAGE and an immunoblot assay, the single-site mutations N112Q, N148Q, and N211Q of the γ2 subunit all resulted in slightly faster migration and thus smaller molecular weight sizes than the WT protein (Fig. 4*B*). Similarly, the single-site mutations N82Q, N111Q, and N137Q of the γ3 subunit also all resulted in a shift toward a smaller molecular size (∼39 kDa band) than the WT protein on SDS-PAGE (Fig. 4*C*). These results are consistent with a loss of glycosylation at these sites.

The double-site mutations of the γ2 subunit, N112/148Q, N112/211Q, and N1148/211Q, and of the γ3 subunit, N82Q/N111Q, N82Q/N137Q, and N111Q/N137Q, all caused a further decrease in molecular size. The triple-site mutants, N112/148/211Q of the γ2 subunit and N82/111/137Q of the γ3 subunit, migrated fastest with the smallest molecular size (Fig. 4, *D* and *E*). The presence of some smaller smear bands in the triple-site mutant of the γ2 subunit (Fig. 4*D*) suggests the possibility of additional posttranslational modifications. Consistently, compared with the triple-site mutational blockade of N-glycosylation in the γ2 subunit, a further downward shift was observed upon removal of the N-linked glycan by treating the cell lysate with PNGase F or by treating the cells with tunicamycin, inhibiting the N-linked glycosylation process (Fig. 4*D*), indicating the presence of additional N-glycosylation on other sites. In contrast, no minor smear bands were observed with the γ3 subunit, and the triple-site mutation produced the same downward shift in molecular size as that obtained with enzymatic treatment (Fig. 4*E*), suggesting the absence of additional N-glycosylation modification. These data suggest that both the γ2 and γ3 subunits are extensively glycosylated at multiple sites in cells.

### Blockade of N-glycosylation reduced the γ1 subunit’s trafficking to the plasma membrane and availability for BK channel modulation

**Figure 5:**
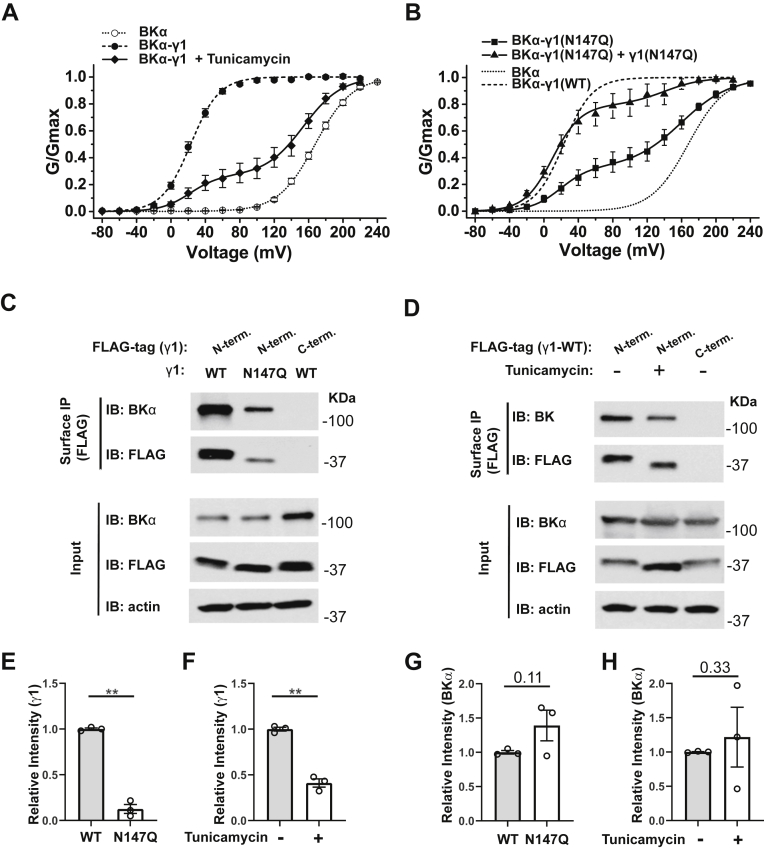
**Effects of N-glycosylation on the channel-modulation function and surface expression of the γ1 subunit.***A*, voltage dependence of BK channel activation for channels formed by cotranslational expression of BKα with γ1-WT in the absence and presence of cell treatment with tunicamycin. *B*, voltage dependence of BK channel activation for channels formed by cotranslational expression of BKα with γ1-N147Q mutant in the absence and presence of supplemental γ1-N147Q overexpression. *C*, surface immunoprecipitation of FLAG-tagged γ1 WT and N147Q coexpressed with BKα. *D*, surface immunoprecipitation of FLAG-tagged γ1 WT coexpressed with BKα in cells that had been treated with or without tunicamycin. *E*, averaged relative intensities of immunoblot staining of surface-immunoprecipitated γ1 WT and N147Q, as shown in *C*. *F*, averaged relative intensities of immunoblot staining of surface-immunoprecipitated γ1 WT from cells that had been treated with or without tunicamycin, as shown in *D*. *G*, averaged relative intensities of immunoblot staining of BKα coimmunoprecipitated with cell surface γ1 WT and N147Q, as shown in *C*. The intensities of surface BKα bands were normalized to those of the corresponding surface γ1 bands. *H*, averaged relative intensities of immunoblot staining of BKα coimmunoprecipitated with cell surface γ1 WT from cells that had been treated with or without tunicamycin, as shown in *D*. The intensities of surface BKα bands were normalized to those of the corresponding surface γ1 bands. Surface immunoprecipitation was performed with rabbit anti-FLAG antibody, and immunoblotting was performed with mouse anti-FLAG antibody. Data are presented as mean ± SEM and n = 3 for data in *E*–*H*. Unpaired Student’s *t* test (two-tailed) was used to calculate *p* values. ∗∗ is for *p* values ≤0.01 (*p* = 0.00093 in *E* and 0.00087 in *F*).

To evaluate the effect of N-glycosylation on the γ1 subunit’s function in BK channel modulation, we performed inside-out patch-clamp recording of HEK-293 cells that cotranslationally expressed the BK channel α subunit and the γ1 WT or N147Q mutant. We found that blockade of protein N-glycosylation in HEK-293 cells by either cell treatment with tunicamycin or N→Q mutation resulted in a great reduction in the portion of BK channels that was modulated by the γ1 subunit (Fig. 5, *A* and *B* and Table S1). In the presence of the cotranslationally expressed γ1 WT protein, all BK channels were fully modulated, in that the G-V relationship could be fitted with a single Boltzmann function (V_1/2_ = 22.5 ± 2.7 mV). However, the tunicamycin treatment and N147Q mutation both caused patch-to-patch variation in BK channel properties. In most excised membrane patches, the G-V relationships needed to be best fitted by two populations of channels, *i.e.*, one population that is fully modulated by the γ1 subunit (V_1/2_ = ∼20 mV) and the other nearly not modulated at all (V_1/2_ ≥ 150 mV) (Fig. 5, *A* and *B* and Table S1), which is a known all-or-none phenomenon of the BK channel modulation by the γ1 subunit (17). On average, ∼70% BK channels were not modulated by the γ1 subunit upon cell treatment by tunicamycin or in the presence of N147Q mutation (Table S1). Thus, we hypothesize that blockade of N-glycosylation at the N147 site markedly reduced the γ1 subunit’s availability in BK channel modulation. To test this hypothesis, we enhanced the availability of the γ1 N147Q mutant protein by greatly overexpressing the mutant protein via cotransfection of the BKα-γ1(N147Q) fusion and the γ1 N147Q alone constructs at a 1:10 ratio (μg:μg) in plasmid DNA. The patch-clamp recording results showed that increasing the expression of the γ1 N147Q mutant could significantly restore the protein’s BK channel modulatory effects by allowing most BK channels (∼80%) to be fully modulated by the γ1 subunit (Fig. 5*B* and Table S1).

To determine how N-glycosylation affected the γ1 subunit’s function, we performed immunoblotting and observed that the total expression of the γ1 protein at the whole-cell level was not significantly changed in the absence and presence of the N147Q mutation (Fig. 5*C*) or cell treatment with tunicamycin (Fig. 5*D*). To determine whether N-glycosylation affected the proteins’ surface expression, we performed cell surface immunoprecipitation of the γ1 subunit. We observed that the amount of immunoprecipitated γ1 protein from the cell surface was decreased by ∼90% in the N147Q mutant (Fig. 5, *C* and *E*) and by ∼60% in tunicamycin-treated cells (Fig. 5, *D* and *F*). To determine whether N-glycosylation affected the association of γ1 with BKα, we compared the immunoblot staining intensities of BKα protein (normalized to that of γ1), coimmunoprecipitated with the cell surface γ1 WT and N147Q mutant protein or with the γ1 WT protein from untreated and tunicamycin-treated cells. We found that blockade or inhibition of N-glycosylation had no significant effect on the association of the cell surface γ1 subunit with the BKα subunit (Fig. 5, *C*, *D*, *G* and *H*). Thus, N-glycosylation affects cell surface expression but has no impact on total expression and the association with the BKα subunit and channel gating-modulation function of the γ1 subunit. This is consistent with our previous report that the TM segment and the short C-terminal tail are the major determinants of the γ subunits’ channel gating-modulation function.

### Blockade of N-glycosylation reduced the γ2 subunit’s total expression and availability for BK channel modulation

**Figure 6:**
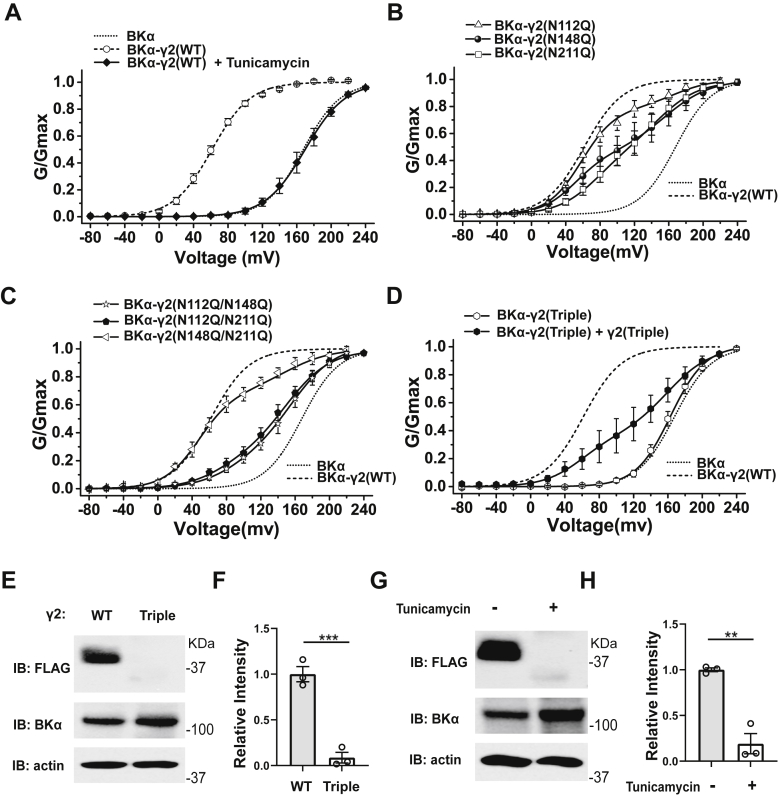
**Effects of N-glycosylation on the channel-modulation function and expression of the γ2 subunit.***A*, voltage dependence of BK channel activation for channels formed by cotranslational expression of BKα with γ2 WT protein in the absence and presence of cell treatment with tunicamycin. *B* and *C*, voltage dependence of BK channel activation for channels formed by cotranslational expression of BKα with the (*B*) γ2 WT, N112Q, N148Q, or N211Q mutant or (*C*) γ2 N112Q/N148Q, N112Q/N211Q, or N148Q/N211Q double-site mutant. *D*, voltage dependence of BK channel activation for channels formed by cotranslational expression of BKα with the γ2 N112Q/N148Q/N211Q triple-site mutant in the absence and presence of supplemental triple-site mutant overexpression. *E*, immunoblot of whole-cell lysates from cells that had been transfected with BKα- and FLAG-tagged γ2 WT or triple-site mutant. *F*, averaged relative intensities of immunoblot staining of γ2 WT and triple-site mutant, as shown in *E*. *G*, immunoblot of whole-cell lysates from cells that had been transfected with BKα- and FLAG-tagged γ2 WT and treated with or without tunicamycin. *H*, averaged relative intensities of immunoblot staining of γ2 WT from cells that had been treated with or without tunicamycin, as shown in *G*. Data are presented as the mean ± SEM and n = 3 for data in *F* and *H*. Unpaired Student’s *t* test (two-tailed) was used to calculate *p* values. ∗∗ and ∗∗∗ are for *p* values ≤0.01 and 0.001, respectively.

To determine the effect of N-glycosylation on the γ2 subunit’s function in BK channel regulation, we measured the voltage dependence of BK channel activation by patch-clamp recording of the BK channels on inside-out membrane patches of HEK-293 cells coexpressing BKα and the γ2 WT and glycosylation-null (N→Q) single-, double-, or triple-site mutants. We observed that treatment of cells with tunicamycin caused full loss of BK channel modulation by the γ2 WT subunit, as the resulting G-V curve is similar to that obtained with the BKα subunit alone (Fig. 6*A*). Similar to that observed with the γ1 subunit, the single-site mutants (N112Q, N148Q, and N211Q) and double-site mutants (N112Q/N148Q, N112Q/N211Q, and N148Q/N211Q) all caused a reduction of the γ2 subunit’s function in BK channel modulation, as indicated by a reduction in the portion of channels with low V_1/2_ (Fig. 6, *B* and *C* and Table S1). This suggests that the γ2 subunit also has the all-or-none modulatory effect on BK channels. Of note, the N148Q/N211Q double-site mutant appeared to be more effective than the single-site mutants N148Q and N211Q in BK channel modulation, suggesting some complex (nonadditive) effect of N-glycosylation on these sites. Similar to the result from cell treatment with tunicamycin (Fig. 6*A*), further blockade of N-glycosylation by triple-site mutation (N112Q/N148Q/N211Q) caused full loss of BK channel modulation by the γ2 subunit (Fig. 6*D* and Table S1). To enhance the availability of the γ2 triple-mutant protein for BK channel modulation, we transfected the cells with the BKα-γ2 fusion and the γ2 alone constructs at a 1:10 ratio (μg:μg) in plasmid DNA. This resulted in significant restoration of the BK channel modulation by the γ2 triple-mutant protein, as ∼36% channels can be fitted with a low V_1/2_ value (∼60 mV) that was close to the maximal effect of the γ2 subunit on shifting the BK channel voltage gating (Fig. 6*D* and Table S1), which further suggests the presence of the all-or-none modulatory effect of the γ2 subunit. These electrophysiological results indicate that the loss of N-glycosylation reduced the availability of the γ2 subunit for BK channel modulation.

To determine how N-glycosylation affected the γ2 subunit’s function, we analyzed the expression of the γ2 subunit WT and the triple-site mutant by immunoblotting. Unexpectedly, the expression of WT protein in tunicamycin-treated cells and the triple-site mutant in untreated cells were barely detectable, unlike the WT protein in untreated cells (Fig. 6, *E*–*H*), suggesting that N-glycosylation is largely required for the expression of the γ2 subunit.

### Blockade of N-glycosylation eliminated the γ3 subunit’s expression on the plasma membrane

**Figure 7:**
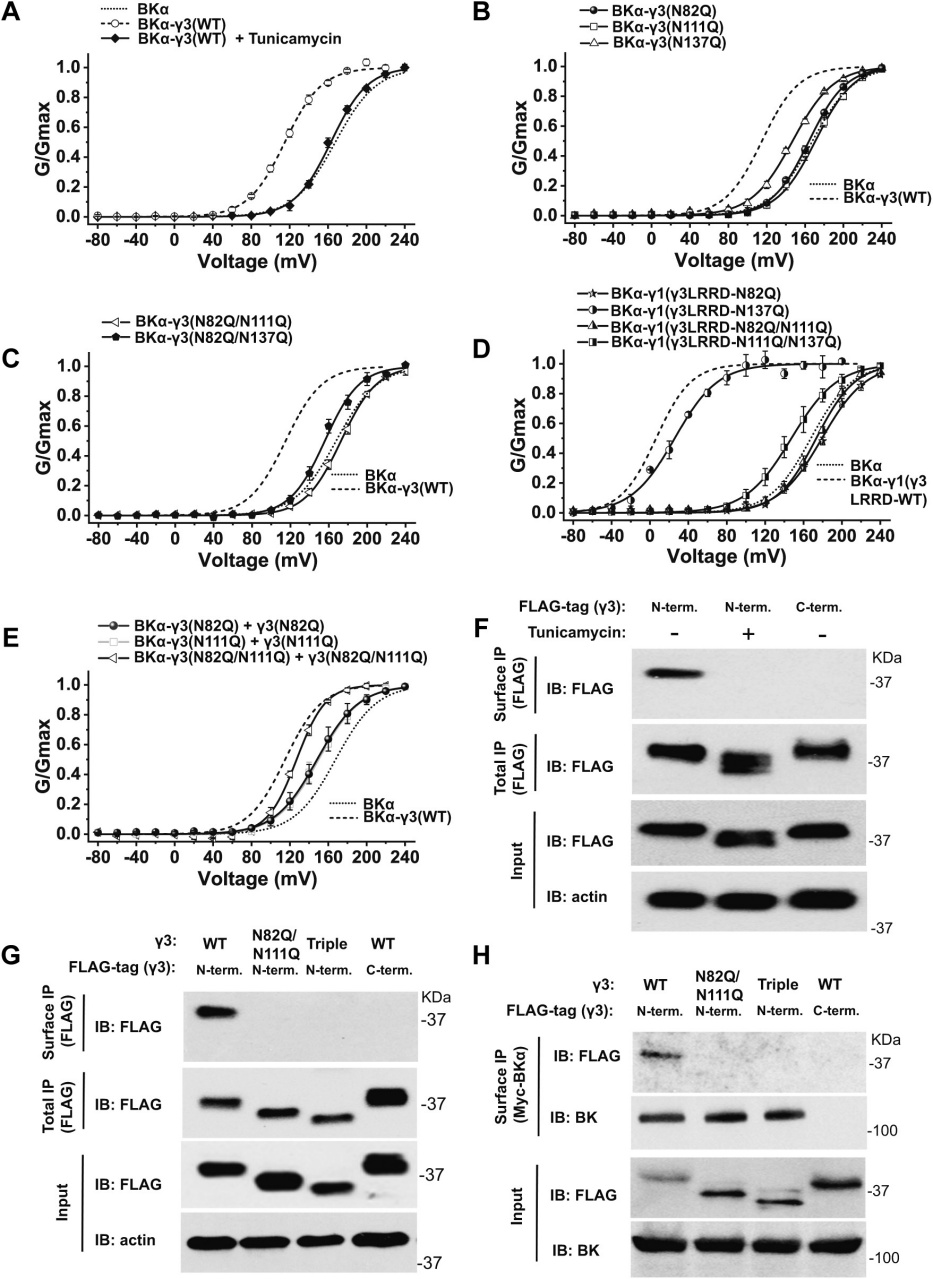
**Effects of N-glycosylation on the channel-modulation function and surface expression of the γ3 subunit.***A*, voltage dependence of BK channel activation for channels formed by cotranslational expression of BKα with γ3 WT protein in the absence and presence of cell treatment with tunicamycin. *B* and *C*, voltage dependence of BK channel activation for channels formed by cotranslational expression of BKα with (*B*) γ3 N82Q, N111Q, or N137Q mutant or (*C*) N82Q/N111Q or N82Q/N137Q double-site mutant. *D*, voltage dependence of BK channel activation for channels formed by cotranslational expression of BKα with γ1(γ3LRRD) chimeric protein’s N82Q, N137Q, N82Q/N111Q, or N111Q/N137Q mutant. *E*, voltage dependence of BK channel activation for channels formed by cotranslational expression of BKα with the γ3 N82Q, N111Q or N82Q/N111Q supplemented with overexpression of the corresponding γ3 mutant. *F*, immunoprecipitation of γ3 WT on the cell surface and whole-cell lysate from cells that had been treated with or without tunicamycin. *G*, immunoprecipitation of γ3 WT, double-site (N82Q/N111Q), and triple-site mutants on the cell surface (surface IP) and whole-cell lysate (total IP). *H*, coimmunoprecipitation of BKα and γ3 WT, double-site (N82Q/N111Q), and triple-site mutants on the cell surface. Immunoprecipitation was performed with a rabbit polyclonal anti-FLAG to pull down the FLAG-tagged γ3 subunit (*F* and *G*) or a rabbit polyclonal anti-Myc to pull down the Myc-tagged BKα subunit (*H*).

Similarly, to determine the effect of N-glycosylation of the γ3 subunit on its BK channel modulation function, we performed inside-out patch-clamp recording of BK channels in HEK-293 cells that cotranslationally expressed BKα and γ3 WT or N-glycosylation site-blockade single-, double-, or triple-site mutants. Our results showed that inhibition of N-glycosylation on γ3 by treating cells with tunicamycin resulted in full loss (V_1/2_ = 160.1 ± 7.4 mV) of the BK channel modulation by γ3 (Fig. 7*A* and Table S1).

Single-site mutation N137Q caused a reduction in BK channel modulation by shifting the V_1/2_ value from 115.0 ± 1.7 (WT) to 149.7 ± 3.8 mV (N137Q), whereas the N82Q and N111Q mutations resulted in full loss of the γ3 subunit’s function in modulating BK channels (Fig. 7*B* and Table S1), as indicated by the resulting V_1/2_ values of 167.2 ± 2.1 (N82Q) and 168.4 ± 3.3 mV (N111Q), which are similar to that of BKα alone (V_1/2_ = 167.7 ± 2.3 mV). As expected, the double (N82Q/N111Q and N82Q/N137Q) mutants also caused a loss of BK channel modulation by the γ3 subunit (Fig. 7*C* and Table S1). We further probed the function of the N-glycosylation on the γ3 LRR domain using a γ1(γ3LRRD) chimeric construct in which the γ1 subunit’s LRR domain is replaced by that of the γ3 subunit and whose function is similar to that of the intact γ1 subunit in BK channel modulation (36). With this construct, the impact of mutational blockade of N-glycosylation sites on the γ3 subunit’s LRR domain was seen more obviously from the large shifts in the voltage dependence of the channel activation. The single and double mutations, N82Q, N82Q/N111Q, and N111Q/N137Q, in the γ1(γ3LRRD) chimeric construct all resulted in full or nearly full loss of the modulatory effect on BK channel, whereas the N137Q mutation alone had only minor effect (Fig. 7*D*). These results are largely consistent with those observed for mutations on the γ3 subunit. We evaluated whether the mutation-induced decrease in the γ3 subunit’s modulatory effect was caused by reduction in the protein’s availability for channel modulation. By increasing the transfected DNA amount of the γ3 mutant relative to BKα (BKα-γ3:γ3 = 1:10 in plasmid weight), we observed enhanced modulatory effects, *i.e.*, shift toward hyperpolarizing direction in the voltage dependence of channel activation, for the three tested mutants, N82Q, N111Q, and N82Q/N111Q (Fig. 7*E* and Table S1), consistent with a reduction in availability of the γ3 subunit for BK channel modulation.

To determine how N-glycosylation affected the γ3 subunit’s availability for channel modulation, we performed immunoprecipitation of the total and cell surface γ3 protein using anti-FLAG antibody to pull down the FLAG-tagged γ3 protein. We observed that the total expression of the γ3 subunit was not significantly different among the WT and the double- (N82Q/N111Q) and triple-site (N82Q/N11Q/N137Q) mutants (Fig. 7, *F* and *G*). However, the surface immunoprecipitation of the N-terminally FLAG-tagged γ3 WT protein showed nearly no detectable expression upon cell treatment with tunicamycin to inhibit N-glycosylation (Fig. 7*F*). Similarly, the surface expression of the γ3 double- (N82Q/N111Q) and triple-site (N82Q/N11Q/N137Q) mutants on the plasma membranes was also largely absent (Fig. 7*G*).

We also performed surface immunoprecipitation of BK channels that were coexpressed with the γ3 WT and double- or triple-site mutant. We used a rabbit anti-Myc antibody to pull down the N-terminally Myc-tagged BKα protein on cell membranes. In this experiment, we used the C-terminally Myc-tagged BK channel α protein as a negative control. The results showed that only N-terminally Myc-tagged BKα protein was enriched, whereas the C-terminally Myc-tagged BKα was absent in the pull-down products (Fig. 7*H*), validating the specificity of the cell surface immunoprecipitation. Consistent with the cell surface immunoprecipitation results of the γ3 subunit (Fig. 7*G*), both double- (N82Q/N111Q) and triple-site (N82Q/N11Q/N137Q) mutants of the γ3 subunit were not coimmunoprecipitated with the cell surface BKα protein (Fig. 7*H*). Thus, we conclude that blockade or inhibition of N-glycosylation fully blocked the trafficking of the γ3 subunit to plasma membranes, making it unavailable for BK channel modulation on the cell surface.

## Discussion

The BK channel auxiliary γ subunits are single TM membrane proteins of ∼300 amino acids in size that form a characteristic LRR domain. LRR domains are known for mediating protein–protein interactions. An increasing number of LRR-containing proteins have been found to be involved in the regulation of ion channel function. However, the exact functions of LRR domains in the BK channel γ subunits and other ion channel regulatory proteins are largely unknown. Our initial mutational analyses indicated that the LRR domain has an essential role in the γ1 subunit function, as deletion of the whole LRR domain and its individual structural units (except LRR5) all caused full loss of the channel-modulation function of the γ1 subunit (16). However, we later found that the differential G-V shifting effects of the γ1–4 subunits on BK channels are predominantly determined by the single TM domain and secondarily by the adjacent positively charged residue cluster on the C-terminal tail (36). Swapping the LRR domain in the γ1 subunit with those of γ2–4 subunits resulted in only 15- to 30-mV shifts in G-V toward the negative voltage direction, suggesting some potential but limited function of the LRR domain in affecting BK channel gating (36). Therefore, questions arose as to whether the LRR domain is indispensable for the γ subunits’ function and the exact functions that the LRR domain has in BK channel regulation by γ subunits. In addition, it is necessary to determine whether the LRR domain is located on the extracellular side, as we predicted (17).

In this study, we systematically investigated the function of the LRR domain in BK channel regulation by γ subunits. We first validated the extracellular location of the LRR domain using an effective surface immunoprecipitation method. By replacing the LRR domain with the extracellular region of the BK β1 subunit and an engineered protein domain of FAPα2, we demonstrated that the LRR domain is not absolutely required for the maximal voltage-gating shifting function of the γ1 subunit; however, interestingly, the LRR domain appears to be necessary for the γ1 subunit to retain the atypical all-or-none phenomenon of BK channel modulation by the γ1 subunit. Given that LRR domains contain canonical N-glycosylation sites and N-glycosylation is well known in the regulation of protein folding, expression, and trafficking, we studied the function of N-glycosylation on the γ1–3 subunits in detail. We found that N-glycosylation plays a critical role in the expression of the γ2 subunit and plasma membrane trafficking of the γ1 and γ3 subunits. Because N-glycosylation exerts its influence via the LRR domains, we concluded that the LRR domains play a key role in regulating the expression or plasma membrane trafficking of the γ subunits. An extracellular domain or region such as an LRR domain is likely to be required for the BK channel γ subunits to be properly folded on the ER membrane and trafficked to the plasma membrane. N-glycosylation facilitates the LRR domain to fulfill such functions.

Although N-glycosylation was found to be absent on the BKα subunit (48), the β subunits were N-glycosylated in their extracellular loops and their biophysical and pharmacological properties were affected by N-glycosylation. Removal of N-glycosylation in the β1 subunit was reported to affect voltage gating, current kinetics, and inhibition by toxins on the extracellular side (49, 50). N-glycosylation of the β2 subunit was reported to affect the channel rectification properties and toxin accessibility to its binding (51). In this study, we found that the effect of N-glycosylation on the γ2 subunit differed from that of the γ1 or γ3 subunits. Of note, the γ2 subunit also differs from the γ1 and γ3 subunits in their C-terminal positively charged clusters in that the former has no influence, whereas the latter potentiate the voltage-gating shifting function of the γ subunits (36). The TM-adjacent positively charged residues on the C-terminal side are known to be important for a proper membrane anchor of the TM segment via the general “positive-inside rule” (52). We previously found that the C-terminal positively charged cluster plays a key role in both the overall function and the G-V shifting capability of the BK channel γ subunits (36, 46). Both the LRR domain and the C-terminal positively charged clusters may act synergistically for proper TM anchoring and plasma membrane expression of the BK channel γ subunits. The presence of less functional positively charged residue clusters in the γ2 subunits might explain why blockade of N-glycosylation in the γ2 subunit caused a defect in total protein expression that is related to folding and degradation, whereas the elimination of N-glycosylation in the γ1 and γ3 subunits mainly affected the later process, trafficking to the plasma membrane. The γ1 subunit contains only 1 N-glycosylation site on the LRR domain but the most abundant positively charged residues (6 Arg) in the cluster. The γ3 subunit contains three N-glycosylation sites, and its cell surface expression appeared to be more sensitive to N-glycosylation, as even single-site blockade can lead to a full loss of the modulatory function and overexpression of the triple-site blockade mutant cannot restore channel modulation.

It is unclear how the absence of an LRR domain affects the all-or-none phenomenon of BK channel modulation by the γ1 subunit. The γ1 subunit can be in complex with the channel in an up to 4:1 ratio (*i.e.*, 4 γ1 per channel or 1 γ1 per BKα subunit) (32, 33, 34). Single channel recording analysis of a β2_1–194_γ1_258–298_ chimeric construct showed that the possession of a single chimeric protein molecule per channel was sufficient to maximally modulate BK channels (32). The loss of the all-or-none phenomenon with the chimeric LRR domain-lacking proteins could be explained by a potential role of the LRR domain in facilitating proper membrane anchoring of the γ1 subunit. In the absence of the LRR domain, the γ1’s single TM segment in some BKα/γ1 complexes might be improperly folded or orientated in the membrane relative to the BKα, which resulted in some changes or variation in individual γ1 protein molecules’ G-V shifting capacity in BK channel modulation.

In this study, we observed that the γ2 subunit also exerts an all-or-none modulatory effect on BK channels, which has not been reported previously. It remains to be determined whether the γ3 subunit also has such a binary modulatory effect on BK channels. The observation that some mutations (Fig. 7, *B*–*E*) caused a shift in the G-V curve rather than a reduction in the portion of fully modulated channels might indicate the lack of an all-or-none effect. However, it could also be caused by a structural perturbation of the LRR domain, whose proper folding is needed for the all-or-none modulatory effect.

Overall, in this study, we identified a multifaceted function of the LRR domains in the BK channel γ subunits. The role of the LRR domain in expression or surface expression explained the previous observation that the LRR domain, and even its individual LRR units, cannot be simply deleted without a full loss of the channel-modulation function. The necessity of the LRR domain in the all-or-none phenomenon of the BK channel modulation by the γ1 subunit suggests a potential role of the LRR domain in regulating BK channel voltage gating, presumably via an influence on the assembly of the γ1 TM domain within the BKα/γ1 channel complex. In principle, these findings could be applicable to the roles of LRR domains in the channel-modulation function of other LRR proteins.

## Experimental procedures

### Expression of BKα and γ proteins in HEK-293 cells

Recombinant cDNA constructs of human BKα, γ1–3 subunits, and mutants were used for heterologous expression in HEK-293 cells. HEK-293 cells (American Type Culture Collection) were transfected with plasmids using PEI MAX (Polysciences, Inc) and subjected to electrophysiological assays 16 to 24 h after transfection. As described (36), BKα-γ fusion cDNA constructs that encode precursor fusion proteins of human BKα on the N-terminal side and γ proteins on the C-terminal side were generated with the pCDNA6 vector and used to facilitate the cotranslational assembly of BKα/γ protein complexes after endogenous cleavage by peptidases at the linker (signal peptide) region in the mature proteins. The γ subunit-alone cDNA constructs, which were also generated with the pCDNA6 vector, were also used to overexpress the γ subunit. The β1_(2–155)_-γ1_(258–298)_ chimeric construct (not fused with BKα) was generated with pCDNA6 vector similarly as reported (46). The FAPα2-γ1_(258–298)_ chimeric construct was generated with pCDNA6 vector using the γ1 (LRRC26) gene sequence and the DNA sequence of the FAPα2 whole protein using plasmid pMFAPα2 (SpectraGenetics, Inc). The plasmids expressing BKα and γ1, β1_(2–155)_-γ1_(258–298)_, or FAPα2-γ1_(258–298)_ were mixed at a 1:1 or 2:1 ratio (whole plasmid weight) for cotransfection into HEK-293 cells.

### Electrophysiology

We performed patch-clamp recording of the BK channel currents in excised inside-out plasma membrane patches of HEK-293 cells with symmetric internal and external solutions of 136 mM KMeSO_3_, 4 mM KCl, and 20 mM Hepes (pH 7.20). The external solution was supplemented with 2 mM MgCl_2_, and the internal solution was supplemented with 5 mM HEDTA without Ca^2+^ to create a virtual Ca^2+^-free solution. Steady-state activation was expressed as the normalized conductance (G/Gmax), calculated from the relative amplitude of the tail currents (deactivation at -120 mV). The voltage of half-maximal activation (V_1/2_) and the equivalent gating charge (z) were obtained by fitting the relations of G/Gmax versus voltage with the single-Boltzmann function G/Gmax = 1/(1 + e^−ZF(V-VH)/RT^), double-Boltzmann function G/Gmax = Pa/(1 + e^−ZaF(V-VHa)/RT^) + (1 − Pa)/(1 + e^−ZbF(V-VHb)/RT^), or triple-Boltzmann function G/Gmax = Pa/(1 + e^−ZaF(V-VHa)/RT^) + Pb/(1 + e^−ZbF(V-VHb)/RT^) + (1 – Pa-Pb)/(1 + e^−ZcF(V-VHc)/RT^), in which VH, Z, P, a-c, F, R, and T denote V_1/2_, gating charge (z), fraction of each component, indication of different components, Faraday constant, gas constant, and Kelvin temperature, respectively. Fitting with a higher-number (4 or 5) component Boltzmann function was not used because of the complexity of the fitting function. Experimental values are reported as means ± SEM.

### Deglycosylation and immunoprecipitation

Enzymatic blockade of N-glycosylation on proteins was done by adding 5 μg/ml tunicamycin to the cell culture for 20 h. Enzymatic removal of N-glycosylation was done by treatment of cell lysate with PNGase F (New England BioLabs Inc) according to the manufacturer’s instruction. Briefly, the cell lysate was mixed with an equal volume of 2× reaction solution containing 100 units/μl PNGase F, 2% NP-40, 10 mM Na_2_PO_3_ (pH7.5) and incubated at 37 °C for 1 to 4 h. Cell lysate was obtained by solubilization of HEK-293 cells with 2% n-Dodecyl-beta-D-maltoside (DDM) in TBS buffer (50 mM Tris and 150 mM NaCl [pH 7.6]) and then removal of the insoluble fractions by centrifugation at 17,000*g* for 10 min. Immunoprecipitation of the total BK channel γ subunit and the α/γ complex was performed similarly as described (53). The cell lysate was incubated (4 °C for 2 h) with immobilized antibody (3–5 μg) that was covalently cross-linked to protein-A agarose beads. After three repetitive washes (10 min each time) with TBS buffer supplemented with 2% DDM, the captured proteins were eluted from beads with either FLAG peptide (100 μg/ml) or an equal volume of 2 × Laemmli SDS-PAGE sample buffer. Protease inhibitor cocktail (Roche) was used throughout the procedure.

For cell surface immunoprecipitation, protein constructs of the N-terminally FLAG-tagged γ1 subunit or N-terminally Myc-tagged α subunit were used to allow specific antibody binding from the extracellular side. About 36 to 48 h after transfection, cells were gently washed 2 times with phosphate-buffered saline (PBS) buffer (Cat# 17-516F, Lonza Bioscience) in dishes and then directly incubated with a rabbit polyclonal anti-FLAG (Cat# F7425, Sigma-Aldrich) or a rabbit polyclonal anti-Myc (Cat# C3956-.2MG, Sigma-Aldrich) antibody (3–5 μg) in PBS for 30 min at ambient temperature. The antibody-associated cells were collected from dishes and lysed with 2% DDM in TBS buffer. After centrifugation at 17,000*g* for 10 min, the solubilized proteins in the supernatant were incubated with protein A agarose beads at 4 °C overnight. The precipitated protein was then washed and eluted following the same procedure described above.

The eluted proteins were separated on 4% to 20% gradient SDS-PAGE gels and transferred to polyvinylidene difluoride membranes. Immunoblotting was performed with mouse monoclonal anti-FLAG (Cat# F7425 and F3165 from Sigma-Aldrich) or anti-BKα (Cat# L6/60 from NeuroMabs) antibody to detect the BKα and γ subunits.

### Molecular dynamic simulation

The initial structural models of LRRC26, LRRC52, LRRC55, and LRRC38 were from the protein structure database of AlphaFold (45). The N-terminal signal peptide sequences were removed at the cleavage sites predicted by the SignalIP-5.0 Server (http://www.cbs.dtu.dk/services/SignalP/). The protein/lipid/solvent systems and input files for molecular dynamic simulation were generated with the CHARMM-GUI webserver (54). The initial structural models were embedded in a lipid bilayer of 1-palmitoyl-2-oleoyl-sn-glycero-3-phosphocholine (POPC) within a water box containing 0.15 M KCl in which the protein charges were neutralized with K^+^ or Cl^−^ ions. The molecular dynamic simulation was carried out with Gromacs 2021 (https://doi.org/10.5281/zenodo.5053220) (55) and CHARMM36m force-field (56) with WYF parameter for cation–pi interactions (57). The system was energy minimized and then equilibrated in six steps using default input scripts for Gromacs generated by the CHARMMGUI webserver. After the equilibration, the systems were simulated for 100 ns with a 2-fs time step. The Nose–Hoover thermostat and a Parrinello–Rahman semi-isotropic pressure control were used to keep the temperature at 303.15 K and the pressure at 1 bar, respectively. A 12-Å cutoff was used to calculate the short-range electrostatic interactions, and the Particle Mesh Ewald summation method was employed to account for the long-range electrostatic interactions.

### Data processing and statistics

The data were processed and plotted with Igor Pro (v5), GraphPad Prism (v8), or OriginLab (v2015 or 2017). Unless indicated, all measurements or repeats were taken with distinct samples or cells. Unpaired Student’s *t* test (two-tailed) was used to calculate *p* values.

## Data availability

All relevant data are contained within this article and the supporting information.

## Supporting information

This article contains supporting information.

## Conflict of interest

The authors declare that they have no conflicts of interest with the contents of this article.
