# Supplementary material for: The leucine-rich repeat domains of BK channel auxiliary γ subunits regulate their expression, trafficking, and channel-modulation functions
Source: J Biol Chem. 2022 Jan 30;298(3):101664. doi: 10.1016/j.jbc.2022.101664 (PMC8892010; doi:10.1016/j.jbc.2022.101664)

Supporting Information

The leucine-rich repeat domains of BK channel auxiliary γ subunits regulate their expression, trafficking, and channel-modulation functions

Guanxing Chen[1], Qin Li[1], Jiusheng Yan[1,2]

[1]Department of Anesthesiology and Perioperative Medicine, The University of Texas MD Anderson Cancer Center, Houston, Texas, USA
[2]Neuroscience and Biochemistry and Cell Biology, The University of Texas MD Anderson Cancer Center UT Health Graduate School of Biomedical Sciences, Houston, Texas, USA

Correspondence and requests for materials should be addressed to J.Y. (jyan1@mdanderson.org).

Table S1 Boltzmann-fit parameters of the voltage-dependent BK channel activation in the presence of auxiliary γ subunit wild-types and mutants.

Expression	Boltzmann fit parameters		
	$V_{1/2}$ (mV)	z	n
BKα alone	167.7±2.3	1.24±0.05	11
+ γ1	22.5±2.7	1.68±0.07	10
+ γ2	61.4±3.0	1.22±0.07	9
+ γ3	115.0±1.7	1.36±0.05	6
+ γ1 + tunicamycin	21.8±4.9 (27%)[a,b]	1.53±0.13[b]	11
	154.0±3.4 (73%)	1.16±0.09	
+ γ1 (N147Q)	18.8±5.8 (32%)[b]	1.53±0.14[b]	11
	159.8±5.1 (68%)	0.90±0.07	
+ γ1 (N147Q) overexpression	10.0±5.4 (79%)	1.57±0.16	8
+ γ2 + tunicamycin	169.2±6.3[b]	1.25±0.09[b]	7
+ γ2 (N112Q)	58.4±1.5 (79%)	1.30±0.08	9
	167 (21%)[b]	1.16±0.24[b]	
+ γ2 (N148Q)	50.7±3.5 (45%)	1.30±0.12	6
	155.0±4.2 (55%)	0.95±0.12	
+ γ2 (N211Q)	75±21 (49%)	1.10±0.13	8
	150±34 (51%)	1.08±0.87	
+ γ2 (N112/148Q)	87±33 (23%)[b]	1.00±0.29[b]	6
	157±11 (77%)	1.00±0.23	
+ γ2 (N112/211Q)	74±23 (23%)[b]	1.06±0.28[b]	12
	150±8 (77%)	1.00±0.15	
+ γ2 (N148/211Q)	48±5 (69%)	1.27±0.14	7
	150±14 (31%)[b]	0.96±0.50[b]	
+ γ2 (N112/148/211Q)	162.4±4.3	1.31±0.04	7
+ γ2(N112/148/211Q) overexpression	58.6±5.7 (36%)	1.18±0.02	7
	160±4 (64%)	1.00±0.08	
+ γ3 + tunicamycin	160.12±7.38	1.45±0.08	5

+ γ3(N82Q)	167.2±2.1	1.21±0.04	6
+ γ3(N111Q)	168.4±3.3	1.26±0.07	4
+ γ3(N137Q)	149.7±3.8	1.07±0.04	5
+ γ3(N82/111Q)	172.7±2.1	1.40±0.08	6
+ γ3(N82/137Q)	155.3±3.8	1.65±0.22	5
+ γ3(N82Q) overexpression	148.6±7.1	1.42±0.09	9
+ γ3(N111Q) overexpression	149.2±5.2	1.24±0.04	8
+ γ3(N82Q/N111Q) overexpression	127.0±1.4	1.68±0.07	3
+ γ1(γ3LRRD-N82Q)	179.3±0.7	1.13±0.02	4
+ γ1(γ3LRRD-N137Q)	26.0±1.7	1.16±0.15	3
+ γ1(γ3LRRD-N82Q/N111Q)	173.0±2.5	1.31±0.15	4
+ γ1(γ3LRRD-N111Q/N137Q)	147.3±7.5	1.14±0.02	4

n values are the number of recorded excised inside-out patches from different HEK-293 cells.

[a] The indicated percentage in parentheses here and elsewhere refers to the portion of the channels' subpopulation that was obtained from a double Boltzmann function fit.

[b] Because of the difficulty in obtaining reliable parameter values from a double Boltzmann function fit for the minor portion (e.g., ≤35%), the estimated values of the $V_{1/2}$ and errors provided here are considered less reliable and used for references only.

Table S2 Double- or triple-Boltzmann global fit parameters of the voltage-dependent BK channel activation in the presence of auxiliary γ subunit wild-types and chimeric constructs.

+ γ1			
	**Double-Boltzmann (Fig. 3B)**		**n**
**$V_{1/2}$ (mV)**	171	31	
**z (e)**	1.04	1.23	
**Fraction**	1	0	11
	0.85	0.15	1
	0.72	0.28	1
	0.65	0.35	1
	0.45	0.55	1
	0.34	0.66	1
	0.21	0.79	1
	0.14	0.86	1
	0	1	10

+ $\beta1_{(2-155)}$-$\gamma1_{(258-298)}$						
	**Double-Boltzmann (Fig. 3C, middle panel)**		**Triple-Boltzmann (Fig. 3C, right panel)**			**n**
**$V_{1/2}$ (mV)**	97	24	166	78	14	
**z (e)**	0.81	1.05	1.23	0.94	1.17	
**Fraction**			1	0	0	11
	1	0	0.28	0.70	0.02	5
	0.87	0.13	0.12	0.87	0.01	5
	0.61	0.39	0.01	0.80	0.19	3
	0.37	0.63	0.01	0.53	0.46	3
	0	1	0	0.15	0.85	4

+ FAPα2-γ1$_{(258-334)}$						
	**Double-Boltzmann (Fig. 3D, middle panel)**		**Triple-Boltzmann (Fig. 3D, right panel)**			**n**
**$V_{1/2}$ (mV)**	135	40	168	99	29	
**z (e)**	0.92	1.20	1.24	1.33	1.52	
**Fraction**			0.98	0.02	0	11
(each fit)	1	0	0.60	0.40	0	5
	0.74	0.26	0.40	0.39	0.21	5
	0.61	0.39	0.14	0.69	0.17	1
	0.37	0.63	0.14	0.38	0.48	1
	0.26	0.74	0.09	0.32	0.59	1
	0.12	0.88	0	0.31	0.69	1
	0	1	0	0.12	0.88	1

Figure S1. Protein sequence alignment and domain/region assignment. Potential *N*-glycosylation sites of Asn residues are shown in red. Cysteine residues that are involved in the formation of disulfide bridges are shown in bold. Hydrophobic residues of Leu, Ile, Val, Phe, Tyr, and Trp are grouped together.

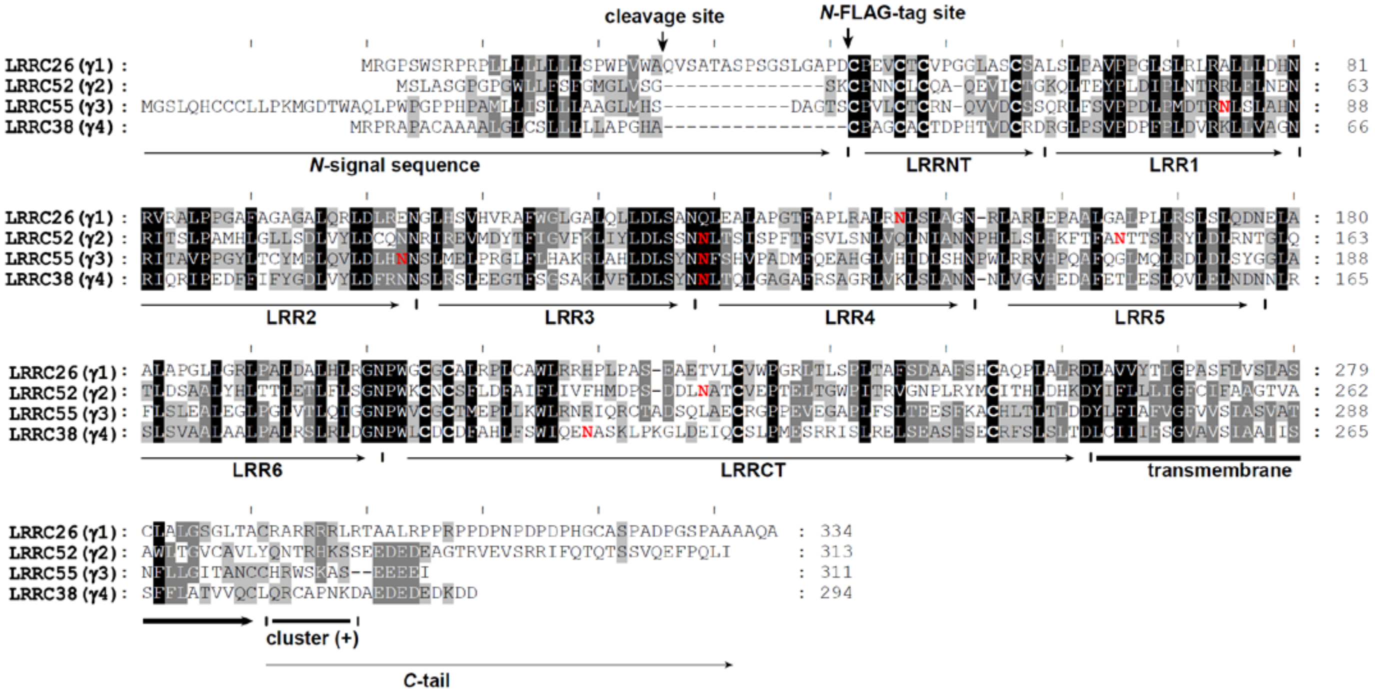

Supplement: Supplemental Figure S1, Tables S1 and S2 [file mmc1.pdf]
